# A systematic review of Sandifer syndrome in children with severe gastroesophageal reflux

**DOI:** 10.1007/s00383-024-05683-3

**Published:** 2024-03-25

**Authors:** Daiki Kato, Hiroo Uchida, Hizuru Amano, Kazuki Yokota, Chiyoe Shirota, Takahisa Tainaka, Wataru Sumida, Satoshi Makita, Akihiro Yasui, Yousuke Gohda, Takuya Maeda, Akinari Hinoki

**Affiliations:** 1 Department of Pediatric Surgery, Nagoya University Graduate School of Medicine, 65 Tsurumai-cho, Showa-ku, Nagoya, 466-8550 Japan; 2 Department of Pediatric Surgery, Aichi Developmental Disability Center Central Hospital, 713-8 Kagiya-cho, Kasugai, 480-0392 Japan; 3 Department of Rare/Intractable Cancer Analysis Research, Nagoya University Graduate School of Medicine, 65 Tsurumai-cho, Showa-ku, Nagoya, 466-8550 Japan

**Keywords:** Sandifer syndrome, Gastroesophageal reflux, Fundoplication, Laparoscopic surgery

## Abstract

**Purpose:**

Sandifer syndrome (SS), which combines gastroesophageal reflux (GER) and a neurological or psychiatric disorder, is an uncommon condition that often takes a long time to diagnosis. We aimed to systematically review available papers regarding SS.

**Methods:**

After presenting our two cases of SS, we systematically reviewed articles published in MEDILINE/PubMed, Cochrane Library, and Web of Science.

**Results:**

The meta-analysis included 54 reported cases and 2 of our own cases. Our results showed that all cases achieved symptom improvement with appropriate treatment for GER. Notably, 19 of the 56 cases exhibited anatomical anomalies, such as hiatal hernia and malrotation. Significantly more patients with than without anatomical anomalies required surgery (*p* < 0.001). However, 23 of the 29 patients without anatomical anomalies (79%) achieved symptom improvement without surgery. Patients who did not undergo surgery had a median (interquartile range) duration to symptom resolution of 1 (1–1) month.

**Conclusion:**

The primary care providers should keep SS in the differential diagnosis of patients presenting with abnormal posturing and no apparent neuromuscular disorders. Fundoplication may be effective especially for patients with anatomical anomalies or those whose symptoms do not improve after more than 1 month with nonsurgical treatment.

## Introduction

Sandifer syndrome (SS) is an uncommon condition characterized by a combination of gastroesophageal reflux (GER) or hiatal hernia and a neurological or psychiatric disorder [1]. SS is associated with abnormal posture and movements of the neck and trunk. Patients with SS present with abnormal posture and involuntary movements that disturb clinicians or parents given that they can mimic seizures [2]. SS is often misdiagnosed as a neurological or musculoskeletal condition. The difficulty in accurately diagnosing this clinical manifestation is that there are often no obvious gastrointestinal symptoms such as abdominal pain or vomiting. This can lead to unnecessary and expensive neurologic examinations such as MRI, EEG, and electromyography. These exams may lead to a missed and delayed diagnosis, and mismanagement. When a patient has abnormal posture or movement without neuromuscular disease, SS should be one of the differential diagnoses. Fortunately, symptoms of SS improve with GER treatments, such as medication and surgery.

Recognizing SS and treating GER will quickly resolve this disease. However, reports on SS have been infrequent, with the diagnosis of SS often taking a long time. Moreover, to the best of our knowledge, only a few systematic review and meta-analysis have been published on this subject. Therefore, the current study first aimed to present our experience with two cases of SS. Second, a systematic review was conducted to evaluate all published studies on patients with SS. Our article on the diagnosis and treatment of patients with SS aimed to help consider SS as an early differential diagnosis in children with these disorders.

## Methods

### Case presentations

#### A 9-year-old boy

**Fig. 1:**
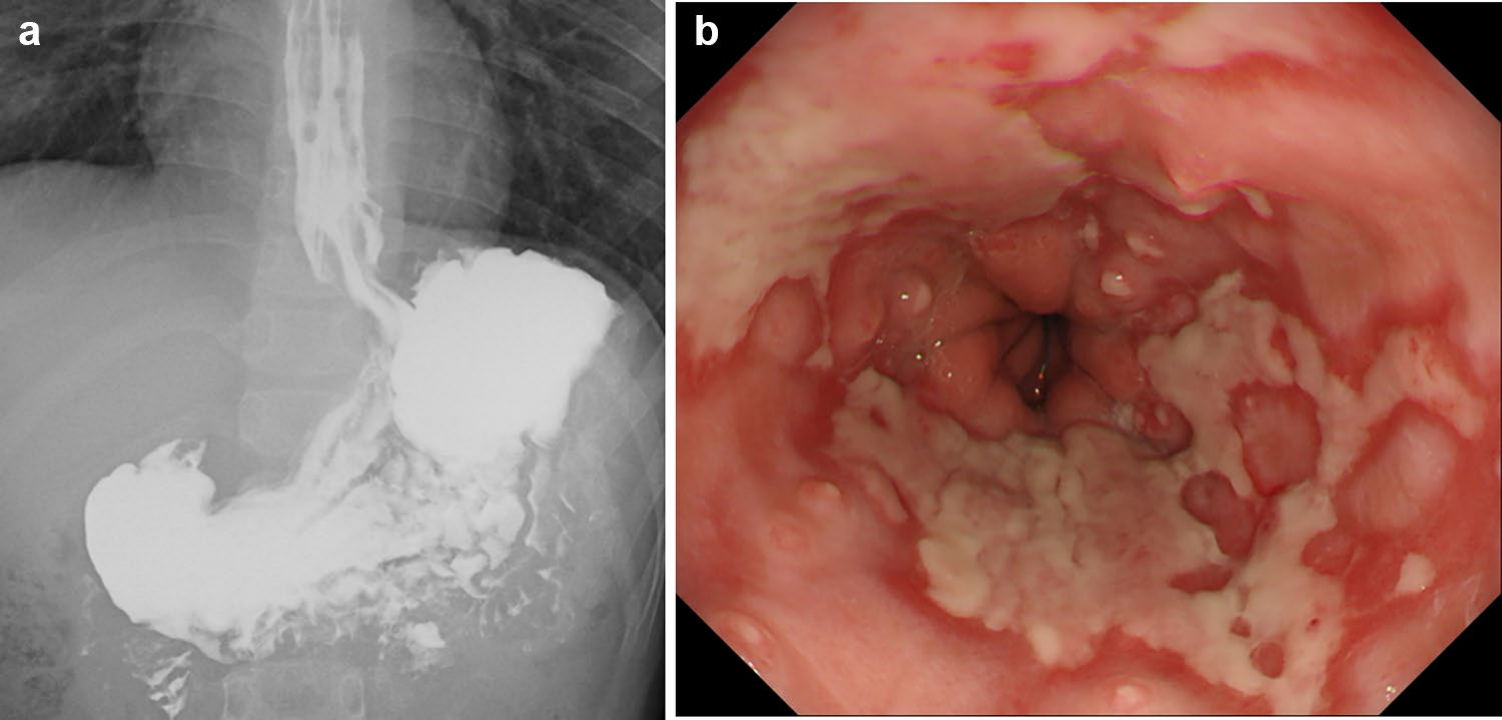
**a** Upper gastrointestinal series showing gastroesophageal reflux. **b** Esophagogastroduodenoscopy showing reflux esophagitis (Los Angeles classification: Grade D) and a hiatal hernia

This case involved a patient who was suffering from vomiting and an abnormal left-leaning posture that started 4 years prior to presentation. He initially sought consultation from a pediatric neurologists. However, when no abnormalities were found on blood examination and head magnetic resonance imaging, he was placed under observation. Unfortunately, his symptoms did not improve, prompting referral to a pediatric psychiatrists 2 years prior to presentation. Electroencephalography and development examinations showed no abnormalities. As such, SS was suspected based on history, for which upper gastrointestinal examinations were performed. Upper gastrointestinal series (UGI) showed GER (Fig. 1a), whereas esophagogastroduodenoscopy (EGD) showed reflux esophagitis (Los Angeles classification: Grade D) and a hiatal hernia (Fig. 1b). Low esophageal pH (< 4) accounted for 19% of the 24-h recording cycle. These findings confirmed that SS was the correct diagnosis.

Given the lack of improvement with proton pump inhibitor (PPI) treatment for 1 month, laparoscopic Toupet fundoplication was performed, which improved the patients symptoms 1 month after surgery. The left-leaning posture disappeared, and the patient did not develop any symptom recurrence for 2 years.

#### A 7-year-old boy

**Fig. 2:**
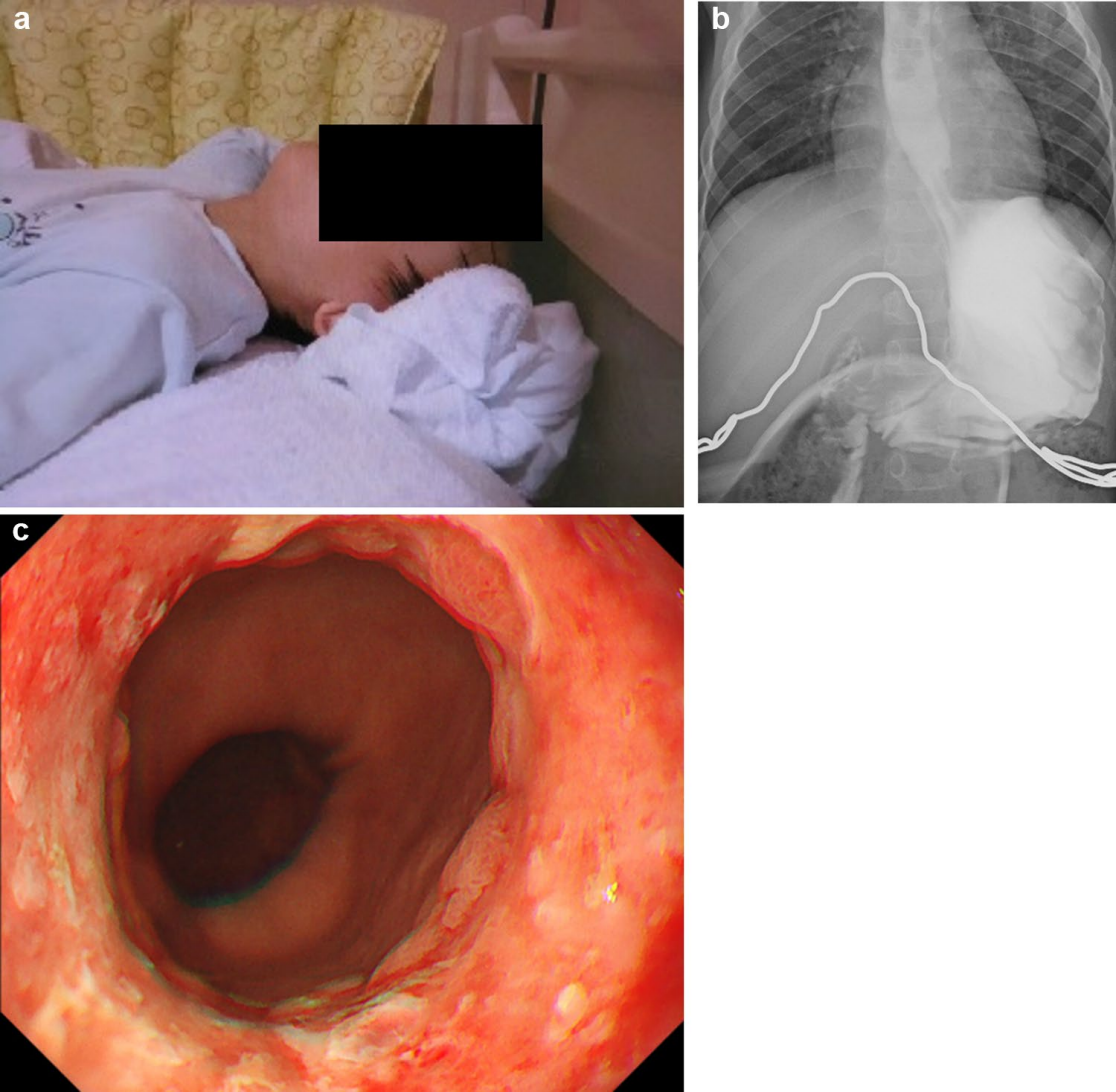
**a** Abnormal posture with backward bending of the neck was observed. **b** Upper gastrointestinal series showing gastroesophageal reflux. **c** Esophagogastroduodenoscopy showing reflux esophagitis (Los Angeles classification: Grade D) and a hiatal hernia

This case involved a patient who was suffering from abnormal posture with backward bending of the neck, violent speech, chronic cough, and vomiting 2 months prior to presentation (Fig. 2a), which prompted him to initially seek consultation from pediatricians. Computed tomography of the head performed in the emergency department of the previous hospital showed no abnormality. The patient was then transferred to our hospital. UGI showed GER (Fig. 2b), and EGD showed reflux esophagitis (Los Angeles classification: Grade D) and hiatal hernia (Fig. 2c). We could not perform low esophageal pH 24-h recording due to his symptom of abnormal posture.

Postural abnormalities temporarily disappeared after initiating PPI treatment 1 month prior to presentation. However, we opted to perform laparoscopic Toupet fundoplication given the recurrence of symptoms despite PPI treatment. At 2 weeks after the surgery, his symptoms improved as shown by the disappearance of postural abnormalities and cessation of voluble speech. No symptom recurrence had been noted for 9 months.

### Systematic review and meta-analysis

We subsequently reviewed articles published in MEDILINE/PubMed, Cochrane Library, and Web of Science using the following combinations of search terms: “Sandifer syndrome” and “gastroesophageal reflux.”

### Selection criteria

Original articles and case reports reporting details regarding patients with SS were included in the analysis. Two reviewers independently scanned the titles and abstracts of the identified articles. The exclusion criteria were as follows: (1) articles not written in English, (2) non-original articles or case reports (meeting abstracts, reviews), (3) studies that did not focus on patients with SS, and (4) studies with insufficient data on patients with SS. This systematic review was conducted based on the PRISMA principles.

### Data extraction

Two reviewers separately collected the following data from the selected articles: the first author, year of publication, study design, sample size, patient characteristics, treatment course, and outcomes. Both reviewers reached a consensus at each stage of the data extraction process.

### Statistical analysis

Continuous variables were presented as medians and interquartile ranges, whereas categorical variables were presented as frequencies and percentages. Fisher’s exact test was used to evaluate categorical variables. *p* values < 0.05 were considered statistically significant.

## Results

**Fig. 3:**
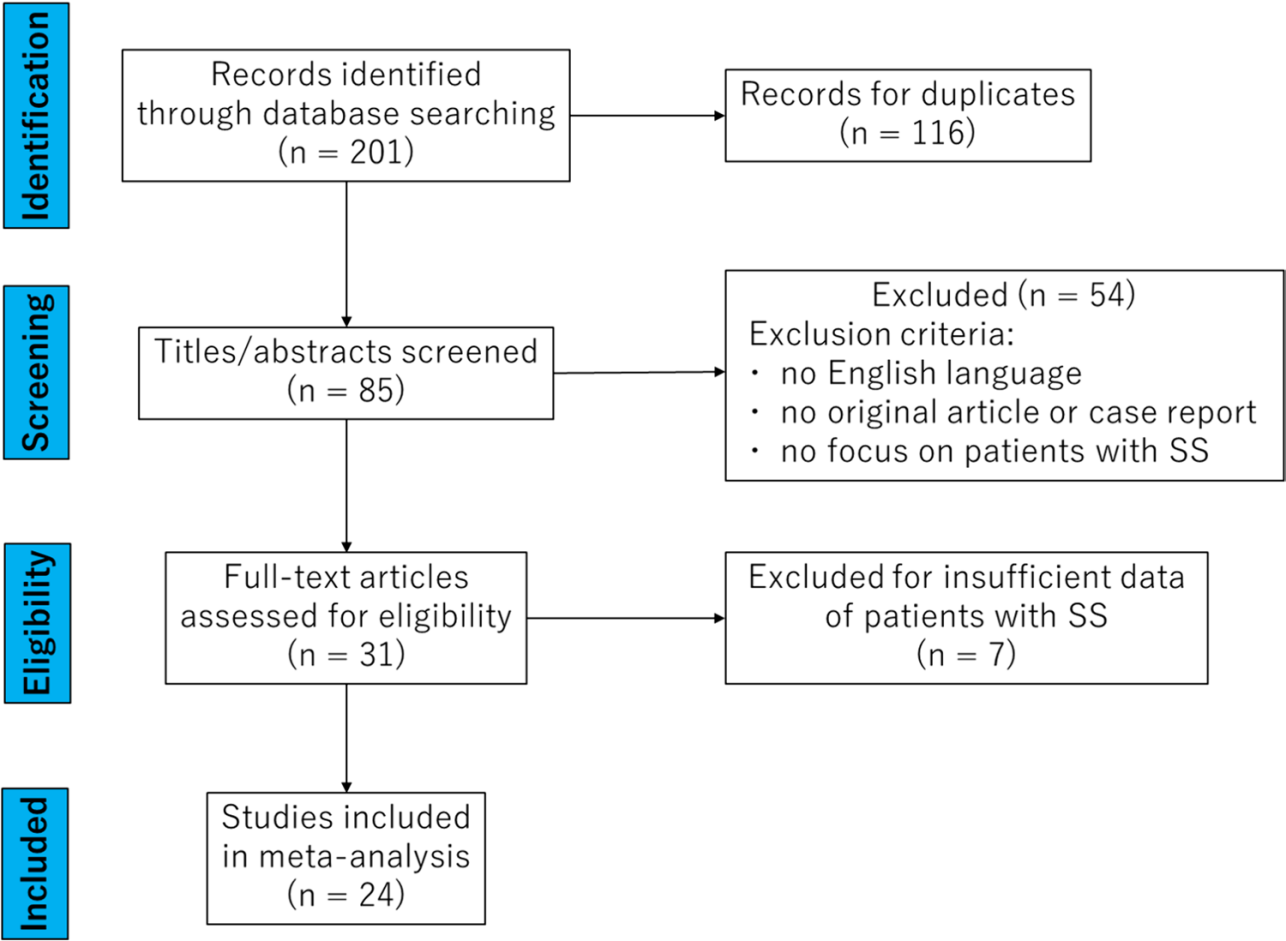
The flow diagram for study inclusion based on the PRISMA 2020 statement

In total, 201 articles were identified via a comprehensive review. After removing 116 duplicates and 61 studies that satisfied the exclusion criteria, 24 articles and 54 cases were ultimately identified (Table 1) [1–24]. Figure 3 shows the flow diagram for study selection.

**Table 1:** Data collected from reported and our cases

Author	Year	Age	Anatomical anomaly	Diagnostic examination	Definitive treatment	Duration to diagnosis (M)	Duration to surgery (M)	Duration to resolution (M)
Shrestha	2021	4y	None	UGI	Drug	N/A	–	1
Sharif	2020	3y	None	UGI, EGD, pH	Drug	N/A	–	N/A
Bamji	2015	3m	None	Clinical history	Drug	2	–	1
		2m	None	UGI	Drug	1	–	1
Nalbantoglu	2008	9m	None	EGD, pH	Diet	2	–	1
Tokuhara	2008	8y	None	UGI, EGD, pH	Surgery	36	3	3
Lehwald	2007	9y	Hiatal hernia	UGI, EGD	Surgery	60	N/A	3
Firat	2007	2y	Malrotation	UGI, pH	Surgery	21	N/A	1
Kabakus	2006	2m	None	Scintigraphy	Drug	5	–	N/A
		4m	None	Scintigraphy	Drug	2	–	N/A
		6m	None	Scintigraphy	Drug	3	–	N/A
		1y	None	Scintigraphy	Drug	8	–	N/A
Corrado	2006	1y	None	UGI, pH	Drug	2	–	1
Frankel	2006	4y	None	UGI, EGD, pH	Surgery	2	30	1
Corrado	2000	15d	None	UGI, pH	Diet	14	–	N/A
Ybarrondo	2000	5y	None	UGI	Surgery	0	12	1
Olguner	1999	5y	None	UGI, pH	Surgery	12	N/A	2
Deskin	1995	2y	None	UGI	Surgery	12	6	3
Gorrotxategi	1995	N/A	N/A	UGI, EGD, pH	Drug	N/A	–	N/A
		N/A	N/A	UGI, EGD, pH	Drug	N/A	–	N/A
		N/A	N/A	UGI, EGD, pH	Drug	N/A	–	N/A
		N/A	N/A	UGI, EGD, pH	Surgery	N/A	N/A	N/A
		N/A	N/A	UGI, EGD, pH	Surgery	N/A	N/A	N/A
		N/A	N/A	UGI, EGD, pH	Surgery	N/A	N/A	N/A
		N/A	N/A	UGI, EGD, pH	Surgery	N/A	N/A	N/A
		N/A	N/A	UGI, EGD, pH	Surgery	N/A	N/A	N/A
Senocak	1993	N/A	Hiatal hernia	N/A	Surgery	N/A	N/A	N/A
Puntis	1989	5y	Hiatal hernia	UGI, pH	Surgery	3	2	3
Nanayakarra	1985	2y	None	UGI, pH, scintigraphy,	Drug	19	–	N/A
		2y	None	UGI, pH	Drug	18	–	1
		2y	None	UGI, pH	Drug	19	–	1
Hadari	1984	13y	Hiatal hernia	UGI	Surgery	N/A	N/A	N/A
Werlin	1980	2w	None	UGI, pH	Drug	0	–	N/A
		2w	None	UGI, pH	Drug	0	–	N/A
		8m	None	UGI, pH	Drug	2	–	1
		3m	None	UGI, pH	Drug	N/A	–	1
		2w	None	UGI, pH	Drug	0	–	N/A
Murphy	1977	8m	Hiatal hernia	UGI	Drug	6	–	1
Bray	1977	3m	None	UGI	Drug	2	–	4
		3m	None	UGI	Drug	2	–	2
		2m	Hiatal hernia	UGI	Drug	3	–	2
		4y	Hiatal hernia	UGI	Surgery	3	N/A	N/A
		5y	Hiatal hernia	UGI	Surgery	30	N/A	1
		5y	None	UGI	Surgery	54	N/A	N/A
		7m	None	UGI	Drug	6	–	N/A
		2m	None	UGI	Drug	1	–	N/A
Sutcliffe	1969	10y	Hiatal hernia	UGI	Surgery	N/A	N/A	N/A
		6y	Hiatal hernia	UGI	Surgery	N/A	N/A	N/A
		4y	Hiatal hernia	UGI	Surgery	N/A	N/A	N/A
Kinsbourne	1964	1y	Hiatal hernia	UGI	Surgery	52	N/A	N/A
		7y	Hiatal hernia	UGI	Surgery	36	N/A	N/A
		4y	Hiatal hernia	UGI	Surgery	47	N/A	N/A
		9y	Hiatal hernia	UGI	Surgery	72	N/A	N/A
		14y	Hiatal hernia	UGI	Surgery	108	N/A	N/A
Kato	2023	9y	Hiatal hernia	UGI, EGD, pH	Surgery	54	1	1
		7	Hiatal hernia	UGI, EGD	Surgery	3	1	1

*UGI* upper gastrointestinal series, *EGD* esophagogastroduodenoscopy, *pH* low esophageal pH 24-h recording

All 54 published cases and both of our own were evaluated via meta-analysis (Table 1). In all cases, symptoms improved with appropriate GER treatment. In terms of anatomical anomaly, there were 18 cases of hiatal hernia, 1 case of malrotation, and 8 cases of no description. Notably, 17/19 (89%) cases with anatomical anomalies required surgery. Patients with anatomical anomalies required significantly more surgeries than did those without anatomical anomalies (*p* < 0.001). However, 23 of the 29 patients without anatomical anomalies (79%) showed symptom improvement without surgery. In both of our cases, surgery promoted early improvement in symptoms of hiatal hernia. Many patients were diagnosed with GER by UGI, EGD, and low esophageal pH 24-h recording. The pH measurement has increased frequency over time and the diagnostic criteria for GER in all cases. The median (interquartile range) age at diagnosis was 2 (0–5) years. The median (interquartile range) duration to diagnosis was 6 (2–28) months. The duration from nonsurgical treatment to surgery was more than 1 month, although only a few articles described this. In patients who did not undergo surgery, the median (interquartile range) duration to resolution of symptom was 1 (1–1) month.

## Discussion

SS, which is named after neurologist Paul Sandifer, had first been reported by Kinsbourne in 1964 after recognizing a dysfunction in the upper gastrointestinal tract with neurological manifestations occurring in children and adolescents [24]. SS consists of an unusual combination of GER and various symptoms such as torticollis, dystonia, and seizures [25, 26]. While the actual incidence of SS remains unknown, estimates place it at probably < 1% of children with GER [27, 28]. Given its lack of recognition, SS is often mistaken for neuromuscular or neuropsychiatric disorders due to limited regarding the same. Available articles have shown that SS takes several months or more to diagnose (Table 1), often resulting in the repetition of unnecessary tests that further delay diagnosis [29].

To date, the etiology of the muscle dystonia in SS remains unclear. It may be related with the diaphragm and neck sharing common innervation. Some authors have postulated the movements and abnormal postures were a learned behavior to relieve abdominal discomfort and improve esophageal motility. It was proved the direct relationship between dystonic movements and low pH, and it revealed the pH in a patient with SS. During 83 episodes of posturing, none of these episodes occurred during a period of pH > 5 for ≥ 30 s[10]. SS could be caused by gastroesophageal reflux. Successful treatment of the underlying GERD led to a complete resolution of the symptoms. Although GER episodes in patients with SS had initially been considered to be induced by postural abnormalities, radiography during the torsion episode suggested that GER was actually worsening [24]. Previous study reported that neurological manifestations were the result of the vagal reflex [10]. However, it does not explain why these postures cannot adopt this position during sleep. The possible pathophysiologic relationship is that GER episodes cause postural abnormalities. Several articles have reported that esophageal motility improved during head tilting as evidence by the increase in esophageal contraction pressure from 47 to 74 mmHg and propagation velocity from 2.5 to 4 cm/s [13, 17]. The symptoms observed in SS are thought to clear gastric acid, suggesting that GER causes postural abnormalities, as supported by the disappearance of symptoms after GER treatment in our cases and in the reported literature.

It is important that GER be resolved when treating SS. Should appropriate nonsurgical treatment fail to improve symptoms, surgery may be necessary. Fundoplication, the primary surgical procedure considered safe for the treatment of GER [30], should always be considered as a possible treatment option for patients with SS. The current study found that significantly more patients with anatomical anomalies required surgery than did those without the same (Table 1). Fundoplication may be more effective than drug or diet therapy in controlling SS should gross anatomical anomalies, such as hiatal hernia and malrotation, be the cause of GER. Moreover, most patients treated with drug or diet improved within 1 month (Table 1). Fundoplication may need to be considered if symptoms do not improve for more than 1 month with nonsurgical treatment.

The primary care providers should keep SS in the differential diagnosis of patients presenting with abnormal posturing and no apparent neuromuscular disorders. Recognition and treatment of GER in patients with SS is key to medical management. The important first step is to suspect SS and obtain a detailed medical history. If symptoms appear immediately after feeding, SS may be present. As the diagnosis of GER is most reliably made by assessing the presence or absence of reflux, pH measurement is recommended in principle. In reported articles, the pH measurement has increased frequency over time (Table 1). The pH measurement is useful as it provides an objective assessment of reflux and clinicians should always consider performing this examination. Treatment should be initiated immediately after confirming GER through upper gastrointestinal examination. Fundoplication may be effective especially for patients with anatomical anomalies or those whose symptoms fail to improve for over 1 month with nonsurgical treatment. Quick detection of subtle symptoms can facilitate early diagnosis and treatment and omission of unnecessary examinations in patients with SS.

Our study has several limitations. First, all eligible studies on patients with SS were case reports and non-randomized in nature. A prospective study may provide additional insights into the diagnosis and outcomes of patients with SS. Second, the sample size was small, suggesting the need for large, high-quality randomized controlled trials in the future. Third, the duration of conservative therapy was unknown although we reviewed articles. If conservative treatment is effective, it may be acceptable to continue treatment as is for long time. How long the effect of conservative treatment lasts is a major issue to be addressed in the future.

## Conclusion

The primary care providers should keep SS in the differential diagnosis of patients presenting with abnormal posturing and no apparent neuromuscular disorders. Prompt treatment of GER may lead to early symptomatic improvement and omission of unnecessary examinations. Fundoplication leads to early symptomatic improvement and may be effective especially for patients with anatomical anomalies or those whose symptoms fail to improve for over 1 month with nonsurgical treatment.

## Data Availability

No datasets were generated or analysed during the current study.
